# Tribological Behavior of Phenolic Resin-Based Friction Composites Filled with Graphite

**DOI:** 10.3390/ma14040742

**Published:** 2021-02-05

**Authors:** En Zhang, Fei Gao, Rong Fu, Yunzhuo Lu, Xiaoming Han, Linlin Su

**Affiliations:** School of Materials Science and Engineering, Dalian Jiaotong University, Dalian 116028, China; enn@djtu.edu.cn (E.Z.); furong@djtu.edu.cn (R.F.); yunzhuohit@gmail.com (Y.L.); hanxm@djtu.edu.cn (X.H.); sulinlin90@163.com (L.S.)

**Keywords:** polymer-matrix composite, graphite, mechanism, solid lubricants

## Abstract

In this paper, the influence of graphite (Gr) on the dry sliding tribological properties of phenolic resin (PF) composites was studied under different sliding speeds of 3.1–47.1 m/s. The wear mechanism was investigated by the observation of the morphology of the transfer layer during the dry sliding process. It was found that the addition of Gr could decrease the friction coefficient and wear rate effectively, and the friction coefficient and wear rate decreased with the increase of Gr content in the range of 10–30 vol.%. The dominant wear mechanisms of PF-based friction composites changed from adhesive wear to fatigue wear (in the form of peeling-off) in the high sliding speed condition after the addition of Gr. The addition of Gr effectively reduced the sensitivity of PF-based friction materials to sliding speeds, and thus enhanced the stability of the friction coefficient. When the content of Gr was above 20 vol.%, the stability of the friction coefficient was relatively steady.

## 1. Introduction

The development of high-speed trains necessitates higher requirements to the brake system. The tribological properties and compositions of friction materials directly affect safe and comfortable brake performance and the ecological environment [1,2,3,4,5,6,7,8,9,10,11]. In recent years, the disc brake system is the most widely used on high-speed trains. The organic brake pads, due to their lighter weight, fewer pollutants and lower energy consumption, have important research value in the development of high-speed train brake systems.

Among organic materials, phenolic resin (PF) with low density, high bonding strength with fillers, good environmental performance and high reliability under severe conditions, has been widely used in braking materials for 250 km/h trains, heavy-duty vehicles and cars etc., and numerous applications [12,13,14,15,16,17,18,19]. It has become one of the important organic brake materials [20,21,22,23,24,25,26,27]. However, with the condition of sliding against the steel braking disc, according to Archard’s friction theory [28] that the removal of lumps from contact areas formed by plastic deformation, high adhesion occurs on the PF. Therefore, the PF shows high wear rate and a large fluctuating friction coefficient, which should be used coupled with the solid lubricant for stable tribological performances [23,29,30,31].

Graphite (Gr), due to its lamellar structure, is an essential solid lubricant component. Gr is found to decrease the friction coefficients and wear rates of resin-based friction materials effectively [1,32,33,34,35,36,37,38,39,40]. For instance, Cho et al. [41] investigated the tribological properties of solid lubricants for automotive brake friction materials. They found that the PF-based friction materials containing Sb_2_S_3_ and Gr improved the friction stability and fade resistance. Zhu et al. [40] reported the friction and wear behavior of furan resin–Gr composite under dry sliding. The results showed that the interaction of furan resin and wear debris of graphite was useful to reduce the wear rate of furan resin–Gr composite. Alajmi et al. [42] investigated the correlation between mechanical properties with specific wear rate and the coefficient of friction of Gr-epoxy composites. They concluded that graphite led to eclectic effects on mechanical properties of graphite/epoxy composites at the same time as affirmative effects on tribological properties of graphite/epoxy composites.

Recent investigations mainly focus on the influence of surface morphology variation on the tribological properties of resin-based multi-component friction materials under different pressures and sliding speeds. The tribological properties of friction material are directly affected by the formation process and composition of transfer layers generated during the sliding contact. With the fillers like glass fibers, aramid pulp, Sb_2_S_3_, MoS_2_ and other components, the study of the transfer layer formation and wear mechanism of Gr in a PF-matrix becomes extremely arduous because of the variation of the transfer layer composition and the interaction between fillers. So far, the effect of Gr on the formation of the transfer layer and wear mechanism is still elusive.

In the present study, we carried out an experiment on the pin-on-disc configurations in a wear-testing machine with a H13 disc sliding against the PF composites. The investigation was on the tribological properties and friction morphology of a series of Gr–PF composites with Gr content in the range of 0–30 vol.% under the different sliding speeds of 3.1–47.1 m/s. The study aims to identify the effect of Gr on the formation of a lubricating layer of Gr–PF composite in the dry sliding process, consequently revealing the wear mechanisms.

## 2. Experimental Procedure

### 2.1. Sample Preparation

The Gr–PF composites were packed in a mold with Gr granules and PF powders. The PF powders containing hexamethylenetetraminse (a curing agent) were used as the matrix. Gr granules with an average size of 300 μm were added to the PF powders at the volume fractions of 0 vol.%, 10 vol.%, 20 vol.%, 25 vol.% and 30 vol.% (denoted as 0Gr, 10Gr, 20Gr, 25Gr, and 30Gr) each as shown in Table 1. Five samples for each volume fraction set were prepared. The ingredients were mixed in a rotary mixer for 1–2 min until they were even, and then kept static for 5–10 min in order to prevent them from wafting out. The mixed raw material powders were loaded into a Φ25 mm × 80 mm mold and hot pressed at 180 °C and 0.23 MPa for 50 min in an oven. After releasing the pressure and taking the specimen out of the mold, they were put into an oven and post-cured at 180 °C for 5 h.

### 2.2. Measurements

The tribological properties of the PF-based friction composites were investigated under dry sliding conditions with pin-on-disc configurations in a wear-testing machine. The friction force generated during the friction test was transformed into the pull force of the sensor, which could show the change curve of the friction coefficient with the friction time on the computer, and the average value of the friction coefficient in the test time was also given. The pin (specimen) was Φ25 mm × 15 mm. A steel disc made of H13 steel of 308 HB was used as the counterpart and the turning radius was 150 mm. At the beginning of the test, the cured pin specimen was pre-ground until it achieved a complete contact with the disc on pin-on-disc tribometer. The surface roughness (Ra) of the pin was 3–6 μm. The contact surface of the disc was polished by hand with grand 800- and 2000-grit abrasive papers, respectively, cleaned with acetone to remove the surface contaminants and dried prior to testing. The contact surface roughness (Ra) of the steel disc was <1.5 μm. The ranges of the sliding speeds were 3.1–47.1 m/s with the contact pressure of 0.51 MPa. The testing experiments were carried out for 5 min for each friction (the ranges of sliding distance are 930–14,130 m). The friction stability was defined as μ_avg_/μ_max_ × 100 %, where μ_avg_ was the mean of dynamic friction coefficient (μ_d_), and μ_max_ was the maximum of μ_d_ [43]. Wear rate was measured as a weight loss method followed by conversion in volume loss using density data. The wear rate is expressed in the form of specific wear rate, K (m^3^/Nm) and calculated as,

K = Δm/utρP(1)

where Δm is weight loss in g, u is sliding speed in m/s, t is sliding time in s, ρ is density in g/m^3^ and P is load in Newton.

The microstructures of the friction surfaces were observed by an optical metallographic microscope (OM), confocal scanning laser microscope and scanning electron microscope (SEM). The parameter statistic of grooves on the worn surface was measured by a confocal scanning laser microscope. The worn surface was investigated with SEM after gold coating and operated at 20 kV.

## 3. Results and Discussion

### 3.1. Material Features

**Figure 1:**
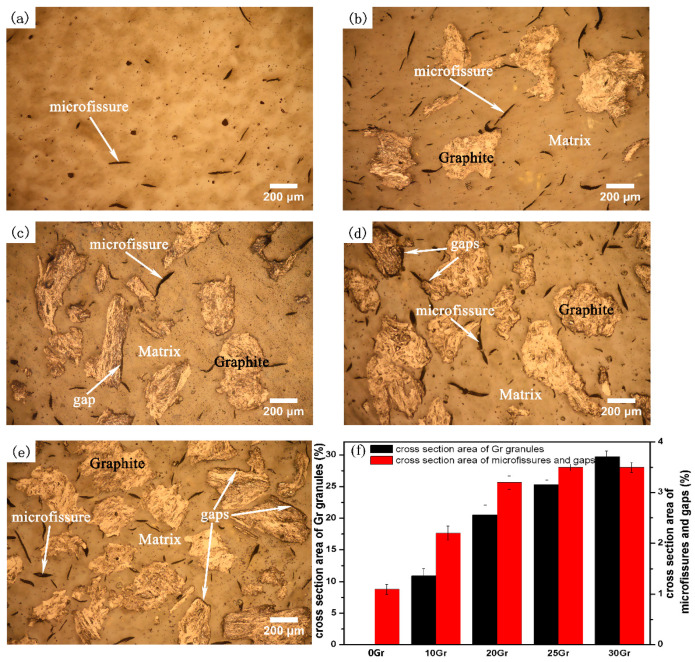
Microstructures of investigated specimens before test: (**a**) graphite (Gr)-free phenolic resin (PF) matrix; (**b**) 10 vol.% Gr/PF; (**c**) 20 vol.% Gr/PF; (**d**) 25 vol.% Gr/PF; (**e**) 30 vol.% Gr/PF, (**f**) column diagram of Gr granules, micro-fissures and gaps’ cross section area.

Figure 1a is the morphology of the raw matrix of PF before test. As can be seen, the surface is dense with few micro-fissures. The micro-fissures are also present in other four specimens compounded with Gr. The micro-fissures may be caused by the volume change during the post-cure processing. It can also be found from Figure 1a that the Gr granules, at the size of 300 to 400 μm, are randomly distributed in the matrix. In addition, as shown in Figure 1b–e, the cross-section area of Gr granules increases with the increasing Gr content (shows in Figure 1f). It can also be noted from Figure 1d,e that gaps appear between the matrix and Gr granules in specimens with higher Gr content. In contrast, the matrix is tightly bound to Gr granules in the specimens with lower Gr content, as presented in Figure 1b,c. The formation of the gaps between matrix and Gr granules is due to the poor interfacial bonding of matrix and Gr granules [44,45].

### 3.2. Friction and Wear Properties

**Figure 2:**
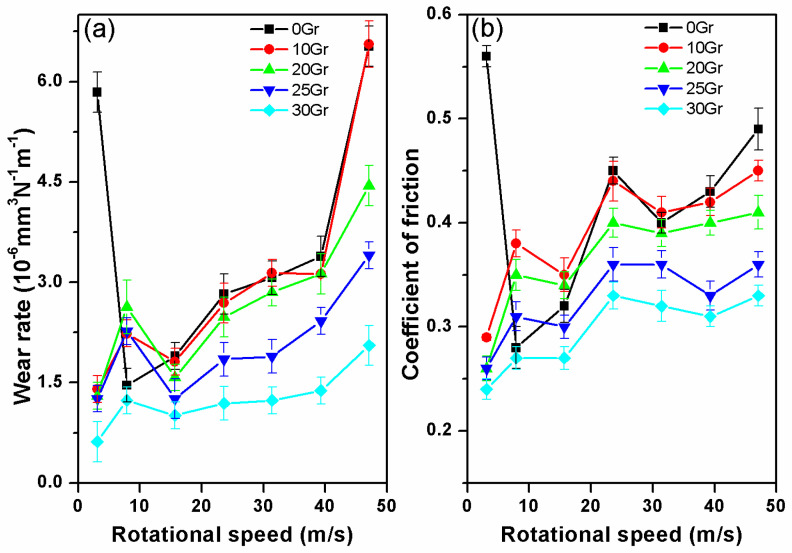
Variations of friction coefficient (**a**) and wear rate (**b**) of the PF-based friction composites with an increase in sliding speed at 0.51 MPa for 5 min.

**Figure A1:**
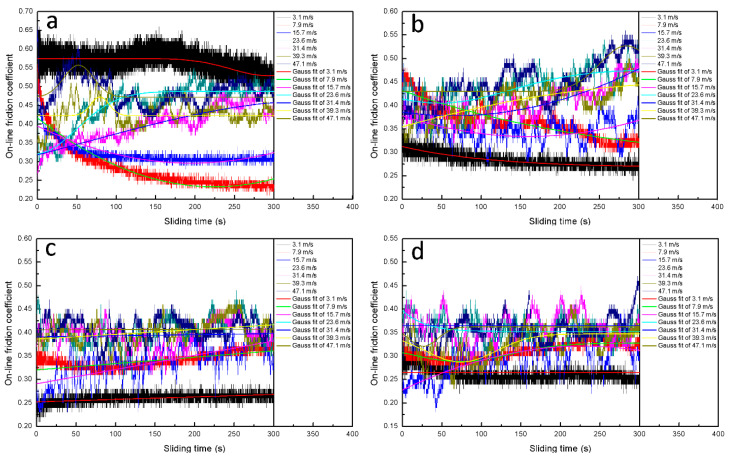

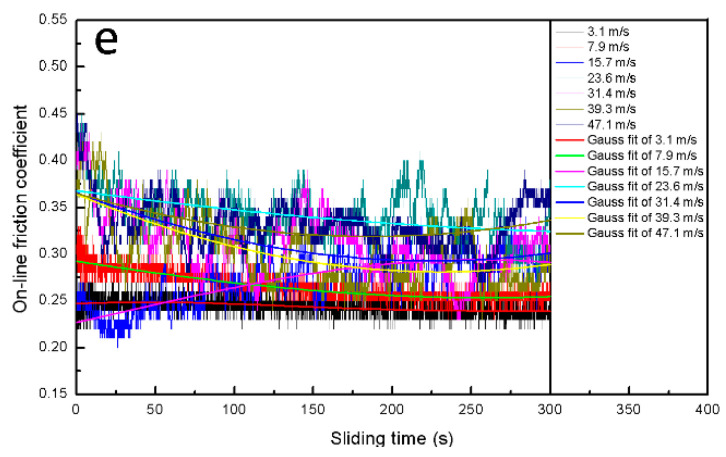
The on-line friction curves of each PF composite: (**a**) 0Gr; (**b**) 10Gr; (**c**) 20Gr; (**d**) 25Gr; (**e**) 30Gr.

Figure 2 shows the variations of the friction coefficients and wear rates of the PF-based friction composites filled with different contents of Gr, sliding against the steel disc at an increasing sliding speed. The on-line friction curves of each PF composite are shown in Appendix A Figure A1. The data for the raw matrix of PF are also presented in Figure 2 for comparison. By contrast with the other four kinds of composite, the PF is sensitive to the speeds and the variations of the friction coefficient. That is, the PF shows a friction coefficient of 0.28 at 7.9 m/s, and then decreases with the increase of speed and obtains the lowest friction coefficient of 0.18 at 15.7 m/s. The friction coefficient suddenly jumps to 0.41 at 23.6 m/s and decreases to 0.37 at 31.4 m/s. After that, the PF shows a positive friction–rotational speed relationship. The wear rate of PF increases smoothly with the increasing speed from 7.9 m/s to 39.3 m/s. However, when the speed is up to 47.1 m/s, the wear rate increases sharply, and is more than twice the average wear rate of PF at different speeds (shown in Figure 2a,b). The fluctuant variations of the curves indicates that the friction performance of raw matrix of PF is unstable with the increasing sliding speeds. Frictional phenomena such as severe noise and vibrations are observed during the friction tests, which correspond to PF composite without friction modifier that have been reported [46].

By contrast with unfilled PF, the PF-based composites with the addition of Gr (specimens 10Gr, 20Gr, 25Gr and 30Gr) show a stable friction–rotational speed relationship. The wear rates of specimens with higher Gr content (25Gr and 30Gr) decrease significantly compared to the PF without solid lubricant fillers. In particular, the specimen 10Gr records a stable decreasing friction coefficient and wear rate when the speed reaches 15.7 m/s. With the speed increasing from 15.7 to 47.1 m/s, the friction coefficient demonstrates fluctuation at 23.6 m/s, and then increases smoothly with the speed. Meanwhile, the wear rate increases steadily during the speed from 15.7 to 39.3 m/s, and then increases sharply when the speed rises to 47.1 m/s like PF (shows in Figure 2b). This phenomenon is consistent with that of some relevant and recent reported papers, in which the friction coefficient and wear rate decreased with the increasing Gr content below 10 vol.% [46,47,48]. The specimen 20Gr has the same friction coefficient of 0.35 from the speed of 7.9 to 15.7 m/s. The friction coefficient increases with the increasing speed from 15.7 to 31.4 m/s, and then reaches stable data between 0.4–0.41 when the speeds exceed 31.4 m/s. The variation of the wear rate curve of specimen 20Gr is similar to that of 10Gr when the speed rises from 7.9 to 39.3 m/s. As the speed reaches 47.1 m/s, the wear rate increases but not so sharply as that of 10Gr. It can be concluded that the specimen 20Gr has lower friction coefficient and shows better wear resistance compared with specimen 10Gr. The variation of the friction coefficient curve of the specimen 25Gr is similar to that of 20Gr but with a lower friction coefficient and the wear resistant. However, 25Gr is better and more stable than the latter. From Figure 2a,b, the friction coefficient and wear rate of the specimen 30Gr are much lower than that of other specimens, and show a minor sensitivity both in friction coefficient and wear rate to the rotational speed within the whole range. In general, with the increase of volume fraction of Gr, the frictional composites show lower friction coefficient and better wear resistance with increasing speed.

**Figure 3:**
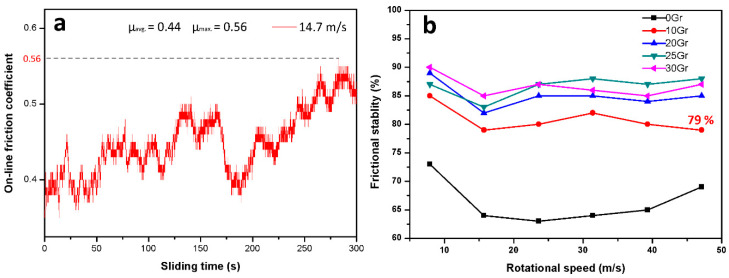
Friction stability of the PF-based friction composites at 0.51 MPa for 5 min: (**a**) the on-line friction curve of 10Gr at 47.1 m/s; (**b**) variations of friction stability of the PF-based friction composites with an increase in rotational speed.

The tribological characteristics of PF-based friction composites are further analyzed in terms of the percentage of friction stability against increasing speed. According to the friction stability equation μ_avg_/μ_max_ × 100%, taking the friction stability of 10Gr at 47.1 m/s for example, the on-line friction curve of 10Gr at 47.1 m/s is shown in Figure 3a, and the friction stability value of 10Gr at 47.1 m/s is 79%. The friction stability of PF-based composites at different sliding speeds is calculated in this way. The results are shown in Figure 3b. The undulating range of the friction stability curve shows the sensitivity of PF-based composites to the increasing speed. In other words, if the curve of a friction composite is flat, the friction stability is rated as low sensitivity to speed [44]. As seen from Figure 3b, the friction stability of PF-based composites filled with Gr is in the range of 79–90%. Meanwhile, the Gr-free PF is in the range of 63–73% and shows unstable friction performance on wear coefficient. The specimens 20Gr, 25Gr and 30Gr with higher content of Gr above 20 vol.% have similar friction stability. However, the specimen 10Gr shows lower friction stability than others. Each of the differences between maximum and minimum friction stability of the Gr–PF composites (10Gr, 20Gr, 25Gr and 30Gr) is in the narrow range of 5–7% and shows low sensitivity to speed.

According to Archard’s theory of adhesive wear, it can be inferred that the raw matrix of PF adheres to the friction disc, and the micro lump could be taken away by the counter disc. With the roughness of the friction surface increasing, the actual contact area between the friction surface and the friction disc increased. Due to the discontinuity of the adhesion point, the friction coefficient appears unstable. When graphite is added as lubricant, the graphite-containing transfer layer which is formatted in dynamic balance is formed on the friction surface. Therefore, the adhesive wear between PF and friction disc is reduced, and the friction stability is improved effectively.

**Figure 4:**
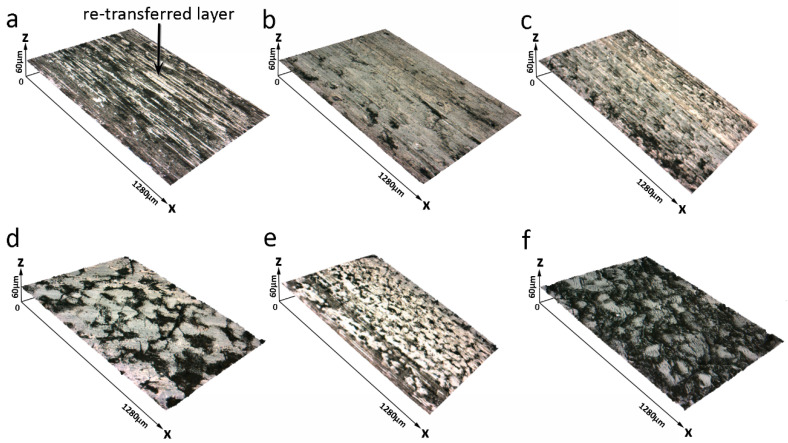
Three-dimensional (3-D) images of friction surface of the PF friction material at different friction speeds at 0.51 MPa: (**a**) 3.1 m/s, (**b**) 7.9 m/s, (**c**) 15.7 m/s, (**d**) 23.6 m/s, (**e**) 31.4 m/s, (**f**) 47.1 m/s.

Figure 4 shows the surface morphology of the Gr-free specimen at different speeds. When the sliding speed is 3.1 m/s, there are many grooves, spalling pits and compacted debris (Figure 4a), which indicate that the main wear mechanism is abrasive wear [10,49,50]. For the PF and counterpart contact directly, strong plowing and asperity contact are responsible for the rough friction surface and high friction coefficient and wear rate (Figure 2). When the speed reaches 7.9 m/s, the friction surface is flat (Figure 4b). There are shallower grooves and less spalling pits. The continuous transfer films which are formed by abrasive particles covers the whole friction surface. Due to the engagement of PF and the counterpart, the large abrasive particles that fall off PF have a relative movement between the friction surfaces, resulting in a ball effect and a decrease in the friction coefficient (Figure 2b). The surface becomes rough when the sliding speed is up to 15.7 m/s (Figure 4c). When the speed increases to 23.6 m/s, no grooves can be seen on the friction surface, and the compacted debris and a large number of spalling pits exist on the friction surface (Figure 4d). The roughness of the worn surface is increased a lot, which explains the sudden increase of the friction coefficient in Figure 2b. At high sliding speed, the shear force between the asperities of the PF and counterpart increases, and the transfer film on the friction surface becomes dense. The transfer films could break under the fatigue of repetitive contact and no contact and hence they peel off in some areas and become abrasive particles. For this process, the main wear mechanism is adhesive wear after the sliding speed of 7.9 m/s. When the speed reaches 31.4 m/s, the worn surface is covered by large amounts of transfer layer (Figure 4e). Because the transfer layer can reduce the frictional force between friction counter face, the friction coefficient decreases accordingly (Figure 2b). There is a large area of spalling pits on the friction surface when the speed reaches 47.1 m/s (Figure 4d). The friction surface is rough (surface roughness (R_a_) is 3.96 μm), and the compacted debris shows on the actual contact areas. On one hand, under the high sliding speed condition, the temperature of the friction surface is high, accelerating the local aging of PF, consequently reducing surface shear tolerance. On the other hand, the severe fatigue makes it difficult for the friction surface to accumulate continuous compacted transfer films, thereby the fragmentation occurs at the actual contact areas, causing high coefficient and wear rate.

It can be seen from Figure 4 that the re-transferred layer is formed on the surface of PF. At the initial stage of friction, the PF adhered on the steel surface, and when the stripped particles are stuck back to the original PF surface, this can be called re-adhesion. After repeated friction, fine particles are formed on the friction surface, and the final transfer is formed. In fact, the transfer layer comes from the re-adhesion from the steel disc, which can be called the re-transferred layer.

**Figure 5:**
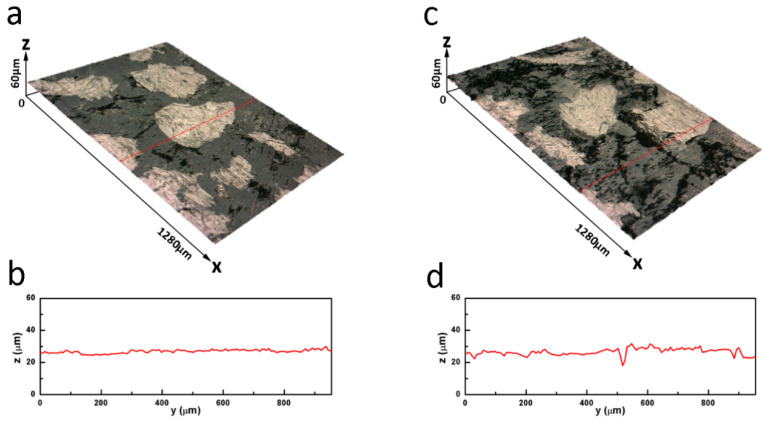
3-D Images of friction surface of the Gr–PF friction composite with different speeds at 0.23 MPa: (**a**) 15.7 m/s, (**b**) profile lines of (**a**), (**c**) 47.1 m/s, (**d**) profile lines of (**c**).

The 3-D morphologies of the friction surface of the Gr–PF friction composite 30Gr at two different sliding speeds are shown in Figure 5a,c. At the speed of 15.7 m/s, the friction surface has a high flatness barring several spalling pits on the friction surface (Figure 5a). The profile lines are average approximately 15.3 μm in width and 1.4 μm in depth (Figure 5b). When the speed reaches 47.1 m/s, because there is a lot of graphite to enhance the lubricating effect, the friction surface is flat (Figure 5c). Although there are some cracks and spalling pits on the friction surface, it is much smoother than that of the Gr-free specimen (Figure 4d).

**Figure 6:**
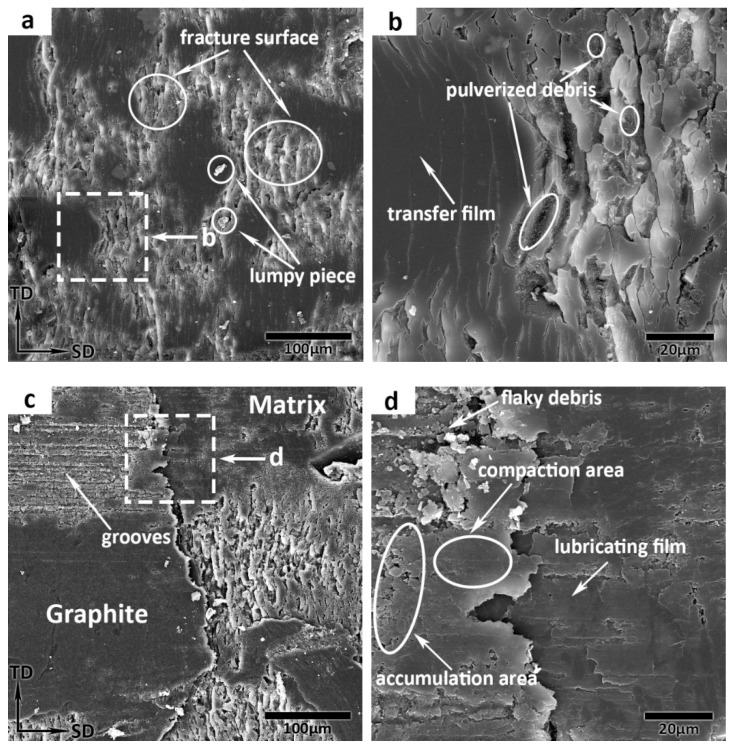
Scanning electron microscope (SEM) images of worn surface at 47.1 m/s: (**a**) Gr-free PF friction composite, (**b**) the zoomed-in views of the rectangular area in (**a**), (**c**) Gr-PF friction composite, (**d**) the zoomed-in views of the rectangular area in (**c**).

The SEM images of the worn surfaces for the samples, as shown in Figure 6, can supply further information for the wear and friction mechanisms. For further information about the wear and friction mechanisms, the magnified SEM images of Figure 4d and Figure 5c are shown in Figure 6a,c. As shown in Figure 6a, part of the friction surface area is fractured and few cut-off lumpy pieces are observed on the worn surface. The friction surface is cracked and fractured, and then the lumpy pieces peel off with the high instantaneous asperity contact pressure and shearing force at the sliding speed of 47.1 m/s. Some lumpy pieces are smashed under the fatigue of repeated rolling and hence they become pulverized debris (see Figure 6b). For this process, the formation of fracture can be attributed to three aspects: degradation accelerated by friction heating, strong local shearing force and plastic deformation. The main wear mechanism is adhesive and it can be inferred that some of the pulverized debris are compacted on the unfractured friction surface and form continuous thin transfer film on local areas.

After incorporating 30 vol.% Gr into the friction composite, the SEM image of Figure 6c shows the Gr–PF boundary on the worn surface and displays milder damage. This result is consistent with the low wear rate and friction coefficient, and mainly attributed to the lubricant performance of Gr. Because the Gr and counterpart contact directly, the friction surface of Gr is scratched by asperities on the counterpart, and grooves and debris form. The magnified image of Figure 6c shows more detail on the worn surface of the Gr–PF boundary and is characterized by flaky debris and lubricating film of Gr (Figure 6d). The above view is also consistent with the results reported by D. Puhan et al. [51]. It is inferred that the flaky debris can be dispersed along the sliding direction on the friction surface, then accumulates in contact plateaus and can be compacted, therefore, as the friction progresses, a continuous lubricating film is formed, and covers the PF worn surface. With the addition of Gr, the fatigue wear is the wear mechanism, and the peeling-off becomes a dominant wearing form of fatigue. Therefore, consistent with the test data shown in Figure 2, the Gr content composites get lower friction coefficient and wear rate than PF composite.

**Figure 7:**
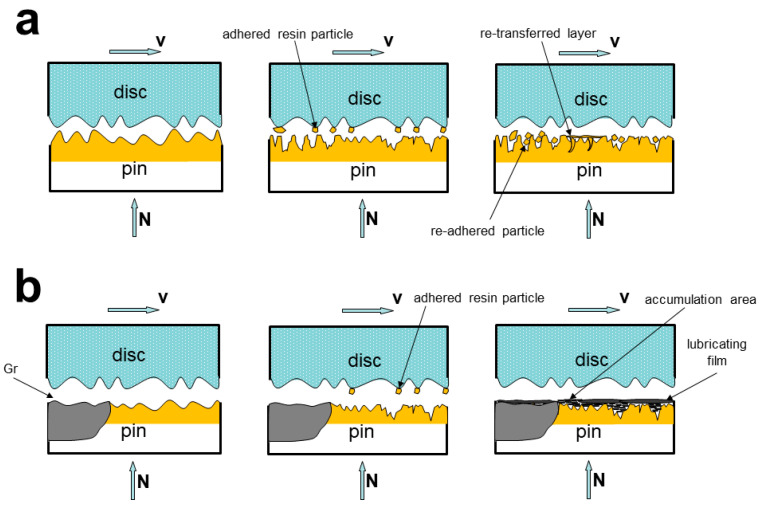
Schematic progress of compact lubricating/transfer film formation: (**a**) PF friction material without the addition of Gr; (**b**) PF friction material with the addition of Gr.

To express the friction mechanism of PF friction materials with the addition of Gr, comparing it with the Gr-free case, the formation mechanisms of the transfer film and lubricating film are listed below. For PF friction materials, their surfaces undergo a process of serious wear and tear, a small amount pulverized debris accumulates on low-lying place and forms a dense re-transfer film (Figure 7a). The rough worn surface which is formed by the severe wear could increase the roughness of the friction surface, so that the actual real contact area increases, and the friction coefficient and wear rate increases (Figure 2). For Gr–PF composite, flaky debris generates on the worn surface. During friction progress, most of the flaky debris are compacted forming smooth and dense lubricating film (Figure 7b). As can be seen from Figure 5 and Figure 6c,d, the worn surface covered by Gr lubricating films is much flatter than the Gr-free PF friction material. The amount of fracture areas of Gr–PF composite is lower than PF friction material. This suggests that the compacted Gr lubricating film decreases the actual contact area, which is conducive to decreasing the friction coefficient and wear rate.

## 4. Conclusions

1. For the Gr-free PF material, there are many grooves and spalling pits on the friction surface at the sliding speed of 3.1 m/s, indicating that the main wear mechanism is abrasive wear. Therefore, the friction coefficient and wear rate are relatively high. When the speed exceeds 7.9 m/s, cracks and shallower grooves appear on the friction surface, and the dominant wear mechanisms change to adhesive wear.
2. The addition of Gr can effectively reduce the sensitivity of PF-based friction materials to sliding speeds, and thus enhance the stability of the friction coefficient. When the content of Gr is above 20 vol.%, the stability of the friction coefficient is relatively stable.
3. With the addition of Gr, the friction coefficient and wear rate decrease effectively compared with the raw matrix of PF. The friction coefficient decreases with the increasing Gr content in the range of 10–30 vol.%.
4. The dominant wear mechanism of PF friction material altered to fatigue wear (in the form of peeling-off) after the addition of Gr. A dense and continuous lubricating layer forms in the spalling pits where the flaky debris piles up and is compacted, which decreases the actual contact area between friction material and the steel disc; therefore, the friction coefficient and wear rate decrease and, consequently, the stability of friction coefficient under different sliding speed is enhanced.

## Figures and Tables

**Table 1:** The composition of friction materials in the volume fraction.

Specimens	PF (vol.%)	Gr (vol.%)
0Gr	100	0
10Gr	90	10
20Gr	80	20
25Gr	75	25
30Gr	70	30

## Data Availability

The data presented in this study are available on request from the corresponding author. The data are not publicly available.

## Appendix A

For the friction coefficient data in Figure 2b are average friction coefficient at each sliding speed, the on-line friction curves of each PF composite are shown in Figure A1 as follows.
